# Storytelling of Young Adults with Chronic Rheumatologic Illnesses: A Pilot Study

**DOI:** 10.3390/healthcare10101979

**Published:** 2022-10-09

**Authors:** Aviya Lanis, Emilee Tu, Malki Peskin, Maryann Melendez, Gabriel Tarshish, Alisha Akinsete, Alicia Hoffman, Kathleen Kenney-Riley, Tamar Rubinstein, Dawn Wahezi

**Affiliations:** 1 Seattle Children’s Hospital, Seattle, WA 98105, USA; 2 Children’s Hospital at Montefiore, Bronx, NY 10467, USA; 3 Colorado Children’s Hospital, Aurora, CO 80045, USA

**Keywords:** narrative medicine, rheumatology, pediatrics

## Abstract

Background: Narrative medicine allows patients to reconstruct medical experiences through written portrayals of perspectives, building a mutual depiction of illness while creating a sense of belonging. This modality has not been previously studied in youth with rheumatologic illnesses, a population with high mental health burden and worse health-related quality of life. We aimed to assess the feasibility of a storytelling intervention in this patient population. Methods: This is a mixed-methods study of 14–21-year-olds with rheumatologic diseases followed in the Bronx, NY. Participants completed an hour-long creative writing session focused on patient experience with chronic disease. Pre- and post-questionnaires assessed patient-reported outcomes, and post-participation video interviews assessed personal experiences through the storytelling session. Results: Thirteen female patients were divided amongst four creative writing sessions. Twelve patients completed pre-study questionnaires and 10 completed post-study questionnaires, with 100% completion of the post-participation interviews. PedsQL surveys showed statistically significant improvement in physical health (*p* < 0.02), and there was no significant difference between pre- and post-scores for any other questionnaires. Interview thematic domains included writing motivation, prior writing experience, illness experience, relating to others, relationship with providers, and support. Conclusion: Creative writing is a feasible and acceptable intervention for youth with rheumatologic illnesses.

## 1. Introduction

Storytelling is a universal form of communication that allows for the expression of a person’s experiences and understanding of their world [1]. Narrative medicine can be described as a subset of storytelling, in which patients can reconstruct their medical experiences through written or oral portrayals of their emotions and self-reflective perspectives [1,2,3,4,5]. By sharing these stories and experiences, research has shown that participants build experiential knowledge and a mutual depiction of the illness while creating a sense of belonging [6]. Currently, there are a variety of available opportunities for group sharing through storytelling for adult patients with chronic illnesses, including self-help groups, support groups, patient associations, patient education and group therapy. Research suggests these group-sharing opportunities can improve quality of life while allowing for a therapeutic venue where participants with chronic illnesses can provide emotional and social support for one another [1,7]. However, limited research has been conducted in group-sharing opportunities in pediatric patients with chronic rheumatologic illnesses, a population with a high risk of poor long-term outcomes [8,9,10].

Children with chronic rheumatologic illnesses are often diagnosed at a critical time of self-development, with the mean age of diagnosis for childhood-onset systemic lupus erythematous, juvenile mixed connective tissue disease, and childhood-onset arthritis to be between 8–13 years of age [11,12,13,14]. With diagnosis at such a critical stage of life, children and adolescents with chronic rheumatologic diseases experience detrimental outcomes including a worse overall health-related quality of life as compared to healthy counterparts [10]. These patients also demonstrate a significant mental health burden, many exhibiting symptoms of depression and anxiety as well as adjustment problems and internalizing symptoms [8,9].

Despite the prevalence of these mental health symptoms and worse health-related quality of life among patients with chronic rheumatologic illnesses, access and delivery of mental health treatment to this population is still lacking [15,16]. Non-medical programs that build emotional resilience may play an important role in providing support that is easily accessed and implemented. They may similarly help prevent poor mental health outcomes in a vulnerable patient population. Storytelling can provide a platform for adolescents to understand their own relationship with their illness as it relates to that of others, as well as positively impact mental, physical and/or psychosocial health.

The current study aims to assess the feasibility of a storytelling intervention, to help inform and develop a larger scale project and to assess changes in symptoms of depression, health-related quality of life and attitudes towards illness. The current study’s goal is to provide a platform for adolescent patients with chronic rheumatologic illnesses to understand the experiences of participants as related to the intervention.

## 2. Materials and Methods

### 2.1. Study Participants

Youth with chronic rheumatologic diseases were enrolled from the pediatric rheumatology clinic at the Children’s Hospital of Montefiore in the Bronx, NY from June to December 2020. Inclusion criteria included patients with chronic autoimmune or auto-inflammatory diseases, ages 14–21. Patients who did not speak and read English were excluded from the study given lack of non-English speaking research staff to conduct sessions in languages other than English. Given restrictions due to the COVID-19 pandemic, consent was obtained over the phone with oral consent, and patients who were recommended by the pediatric rheumatology team were approached. Criteria provided to providers who made recommendations included the above inclusion and exclusion criteria.

Participants engaged in a 1 h virtual-platform-based creative writing session (through Zoom), oriented around patient diagnosis and patient experience with their chronic rheumatologic disease. Attendees at this session included participants in the study, a physician researcher, a nurse and a creative writing expert. The creative writing expert guided each session with a writing warm-up, reading of a healthcare-related poem, and a prompt for participants to write a poem of their own related to a healthcare encounter. Participants were encouraged to share their poems with one another prior to completion of the session. Following completion of the session, participants were provided with anonymous copies of the poems written during each of the sessions.

To assess feasibility of studying such a storytelling intervention, numbers of individuals approached, individuals who agreed to participate, and individuals who completed the program were documented. Additionally, percentages of questionnaires and post-participation interviews completed were documented. Given that the current study is a feasibility study with qualitative interviews, the primary hypothesis was that the study would be feasible, where an expected 80% of all recruited participants were expected to complete all surveys as well as the post-interview portion of the study. For acceptability of the intervention, it was presumed that at least 80% of participants would participate in future sessions as endorsed on the post-participation interviews.

### 2.2. Study Questionnaires

Pre- and post-questionnaires were completed to assess patient-reported outcomes around quality of life and physical and mental health for pediatric patients with chronic rheumatologic diseases. Participants also provided demographic data on self-identified gender, ethnicity, race, and preferred language spoken by patient. Questionnaires included Pediatric Quality of Life Inventory (PedsQL), Pediatric Symptom Checklist-17 (PSC-17), Patient Health Questionnaire-9 (PHQ-9), and the Child Attitude Toward Illness Scale (CATIS). The PedsQL is a 23-item scale created to assess health-related quality of life, which takes approximately 5 min to complete, and has been shown to be an effective measure of health-related quality of life in children and adolescents with rheumatologic diseases [17]. The PSC-17 is a 17-item scale that assesses internalizing and externalizing behaviors, which takes approximately 5 min to complete, and has been shown to be an effective measure of children’s psychosocial functioning [18]. PHQ-9 is a 9-item scale that assesses depression, which takes approximately 3–5 min to complete, and has been shown to have similar sensitivity and specificity as compared to the adult population [19]. Lastly, the CATIS is a 13-item scale measuring illness attitudes within pediatric chronic illness, which takes approximately 5 min to complete and has been shown to be a sound self-report measure of illness attitude within pediatric patients who have chronic illnesses [20]. These questionnaires were completed within a month prior to participation for the pre-participation questionnaires, and between 0–2 months following completion of the narrative medicine intervention. Questionnaires were emailed to participants and participants were asked to email back the completed questionnaires to a HIPAA-compliant email.

### 2.3. Qualitative Interviews

Within four weeks following the creative writing session, participants were contacted over the phone to assess personal reflections about participating in the storytelling session. Open-ended interview questions were agreed upon among researchers prior to the interviews and focused on how the experience made patients feel emotionally, how the experience impacted their feelings with regard to others, their feelings regarding their disease, and their feelings regarding communicating about their disease. Interview questions also addressed overall satisfaction with the intervention, comfort levels of completing pre- and post-surveys, feedback on ways to improve the intervention, as well as participant interest in partaking in future sessions. Interviews lasted between 15 and 40 min, without a specific time limit on the interview completion.

Phone interviews were recorded with participant approval and transcribed for qualitative analysis. Post-participation interviews were then reviewed by three independent researchers (TR, MP, ET) using qualitative software (Dedoose Version 7.0.23, web application for managing, analyzing, and presenting qualitative and mixed method research data (2016). Los Angeles, CA, USA: SocioCultural Research Consultants, LLC., www.dedoose.com) for coding and thematic analysis.

### 2.4. Statistical Analysis

Descriptive analyses were used for patient demographics. Surveys completed pre- and post-intervention were assessed for differences with a Wilcoxon signed-rank test given data was not normally distributed. Statistical analyses were conducted using STATA 14.2 (StataCorp., College Station, TX, USA).

Given that the current study is a feasibility study, process measures included participation rate, ability to complete pre- and post-participation surveys, and assessment of feedback on the experience.

### 2.5. Qualitative Analysis

Qualitative data was analyzed using the framework method approach, previously shown to be a systematic and flexible approach in multi-disciplinary research [21]. The framework method approach involves identification of commonalities and differences in qualitative data before focusing on the relationship between different parts of the data. This allows understanding of descriptive and explanatory conclusions clustered around themes. For the purposes of this study, the interviews were recorded and transcribed into narrative format. Using the framework method approach, three researchers thoroughly read the transcripts and listened to the audio-recorded interviews to become familiar with the data set. Researchers identified codes and themes that demonstrated participants’ experiences and perceptions of the storytelling experience. These themes and codes were established through group discussion of elicited themes by researchers through an iterative process as guided by the framework method approach. These three researchers participated in thematic and content analysis to identify common concepts, patterns and themes so as to obtain meaningful information about the qualitative experiences of participants. Descriptive analysis was used to identify recurrent themes. Codes and categories were identified and constant comparison occurred with the three researchers until there was agreement on final categories and themes.

## 3. Results

Of the 28 patients who were approached, 13 self-reported female participants were recruited, with ages of recruited individuals ranging from 14–21 years. Participants were divided amongst four creative writing sessions, with groups ranging from two to six participants per session. Diseases included lupus (seven), juvenile dermatomyositis (two), systemic sclerosis (one), juvenile idiopathic arthritis (two), polyarteritis nodosa (one), and amplified musculoskeletal pain syndrome (one). Twelve of the thirteen patients completed pre-study questionnaires (92%) and ten completed post-study questionnaires (77%), with 100% completion of the post-participation interviews. Among the 12 participants who completed demographic questionnaires, 3 (25%) reported they were bilingual in English and Spanish and 4 (33.3%) self-identified as Hispanic/Latino. Self-identified race included 6 (46%) Black/African American, 2 (15%) white, 1 (8%) American Indian/Alaska Native, and 2 (15%) Other, with the remainder who did not self-identify race.

Of the 15 patients who were approached but did not participate, 12 chose not to participate for a mix of reasons including not enough time to participate, over-commitment to other research projects, or lack of interest in writing. One patient, who initially consented to participation, retracted consent prior to participation for an unclear reason. Two additional patients wanted to participate but scheduling constraints prevented their participation. These patients were all female, including eight with lupus, four with juvenile idiopathic arthritis, two with sarcoidosis and one with anti-synthetase syndrome.

PedsQL surveys showed a statistically significant improvement in physical health, with pre-participation median 64.95 [IQR 56.25–79.95] and post-participation median 78.15 [IQR 62.5–93.8] (*p* < 0.02), where higher scores indicate better health-related quality of life. There were no significant differences between pre- and post-scores for any of the other questionnaires (Table 1). For interpretation of scoring, a PedsQL score of 80–100 have good quality of life, scores of 60–80 have intermediate quality of life, and scores below 60 are considered to have poor quality of life as related to physical and emotional health. A PSC-17 score of 15 or higher suggests the presence of significant behavioral or emotional problems. For the PHQ-9, a score of 0–4 points reflects normal or minimal depression, 5–9 points indicates mild depression, 10–14 points indicates moderate depression, and 15 or more points is concerning for severe depression. For the CATIS, scores range from 13 to 65 where a higher score indicates a more positive attitude towards the condition.

### 3.1. Qualitative Interview Analysis

Post-participation interview analysis showed thematic domains of writing motivation, prior writing experience, relating to others, illness experience, and support. Participants also spoke about their experience around the writing workshop and provided feedback for future sessions.

#### 3.1.1. Writing Motivation

Participants described using writing as a form of coping, explaining, “I was just in a lot of…mental pain [and] physical pain. It was just a whole lot for me…[and the doctor] recommended [writing] to me”. Another explained, “I don’t know how to express myself…talking to people…so I just [write] everything down”.

Several also referred to the enjoyment they experience when writing, expressing, “I just spewed out all these words, and I felt so good”.

Some were able to find order and meaning through writing: “it really helped me remember times that I did speak up about my disease, and that…reminded me that I should do it as often as I can”.

Participants spoke about the freedom of expression they felt with writing, explaining, “it makes me feel free”. Another participant said it, “helped me be a little bit more descriptive [of] how I explained things”, and another expressed, “if I could do this with strangers, I should be able to do [it] with…my friends and family”.

When asked about what motivated participation in the creative writing session, one participant explained, “It’s not every day I get to meet someone that has something similar [to] me, and hearing [about the other participants] makes me feel like I’m not alone in this world. There’s other people that I don’t even know about”. Another reported appreciation for, “just getting the chance to just share our thoughts and just bond with one another”, and yet another explained, “they have what I have so they understand me and I understand them”.

#### 3.1.2. Prior Writing Experience

When addressing prior writing experience, the primary source for most participants was through school. Several had participated in creative writing classes or more intensive writing outside of school, while two participants had not engaged in prior writing experiences. One expressed that, “whenever you feel this way, just write and…whenever you’re confused, or your thoughts are not really organized, just write it”.

#### 3.1.3. Relating to Others

When asked about whether or not their relationship with their providers had changed after participating in the creative writing session, some felt they had no change in their relationship while others expressed feeling greater ease discussing their disease with their doctor. They also shared surprise that the participants at the session, “had a lot in common, more than I thought we would have”. That participant went on to identify common feelings of pain and shared experiences of requiring infusions and medicines such as prednisone, explaining, “even though we all have different sicknesses, we’re all going through the same thing”. Participants explained they felt a different relationship with other patients as well by engaging in the creative writing sessions, as some had never met other patients with rheumatologic illnesses prior to participating in the session. Participants expressed appreciation for the opportunity to, “express…ourselves,…share our thoughts and just bond with one another”. Another went on to explain, “talking to other people who were going through similar things just kind of helped because I knew that they would understand”. This relationship with other patients was in contrast to reported relationships with peers, where many reported a sense of disconnect from peers with regard to physical abilities and ability to discuss rheumatologic diseases. Overall, participants reported a positive impact of the experience on their relationship with their disease, with one participant explaining, “I feel a little bit more positive…I was thinking on a negative side before”.

#### 3.1.4. Illness Experience

During the post-participation interviews, participants were candid with regard to their illness experience, explaining, “when I was first diagnosed [it] felt like a…complete burden”. One participant described her fatigue as “a tired that doesn’t let you sleep”. Another explained, “I hated pills—I still hate pills actually—[and] I didn’t like going in and out [of] the hospital”. Others expressed frustration with frequency of medical visits explaining, “going to the doctor…every 3 months, or sometimes every month depend[ing on] how the lab comes out…can be annoying”. Many addressed challenges that they experienced, including a feeling of isolation where, “I felt like I couldn’t do stuff that other kids were doing”. Several patients discussed the external perception of the individual’s illness, with one participant explaining, “they start feeling sorry for you and you’re just like…I don’t need that”.

When reflecting on how their relationship with their illness has changed over time, several suggested that they found a uniqueness to having an illness, and one explained, “I feel special…I don’t feel like it’s [something] to be ashamed of anymore”. One explained, “I think I was just born with a big voice. However, I think that having my disease helped me find…an even bigger voice inside of me”. Still others endorsed a sense of resilience and overcoming, expressing, “the more time passes, the more I’m more thankful for lupus”. Another explained, “I feel like my illness doesn’t define me, I feel like I can go throughout the day without having to be like, wow I’m really sick. I feel like it’s…a push back but also a push forward”.

#### 3.1.5. Support

Many participants identified a wide variety of support that helped them cope with their illness, including arts, family, therapy, teachers, friends, and religion, as well as other patients with rheumatic diseases. One participant also identified an online community as a support in coping through disease-specific communities and Reddit, while two other participants found support in one another after connecting on Instagram following completion of the creative writing session. Another identified the healthcare team as a source of support, explaining when, “my doctor…supports me telling me that taking the medicine is good, that helps me and I start taking it”. In sharing about how participation in the session changed her ability to cope with her disease, one participant explained, “it’s helped me change my thinking about this disease, because since we got to share with…other people, I see it as that though I have it, it doesn’t stop me from doing anything else”.

Interestingly, the COVID pandemic arose as one of the discussion topics for four of the participants. One participant explained, “my immune system could be a little weak sometimes…[and] I have to be…extra careful…because I can catch it at any moment at any time”. Another explained, “[I] limit my contact with people, try not to…go outside too much [and] make sure that I have protective gear on when I go out”. One participant discussed the impact that hydroxychloroquine shortage had on her early on in the pandemic, explaining, “my mom wanted to make sure I got it and thankfully they had it [at] the pharmacy…I was…making sure I [had] enough and making sure I [got] the refill...before I [ran] out”. Another participant identified the group session as a reprieve in the pandemic, explaining, “Since the pandemic started, I’ve been feeling less motivated. I haven’t been talking to…people often. So…to be in the group session, talk to other people who have similar…diseases as me, it was really…comforting, made me feel a lot better”.

When discussing potential feedback for future sessions, 12 of the 13 participants stated they would participate in future sessions. Some suggested shortening the length of questionnaires. Additionally, there were some logistical suggestions including considering longer creative writing sessions. Five of the participants suggested having more than one session in the future. One pointed out that not all participants had cameras on during the session, and suggesting making cameras a requirement for virtual participation. Finally, one participant suggested including her primary rheumatologist in the session, expressing, “she’d get to know a little bit more about me”.

## 4. Discussion

Creative writing has long been used within the psychiatric community as a means of healing for those with significant mental health burden [22]. However, only recently have these methods been more regularly implemented for individuals with chronic disease, who notably may be suffering a concurrent mental health burden. A literature review of research from 1995 through 2007 shows there has been an increase in the implementation of therapies such as music engagement, arts therapy, movement-based creative expression and expressive writing as a means of addressing this mental health burden among patients with chronic disease. Particularly focusing on expressive writing, research to date has shown that patients with chronic illnesses experience improvement in physical health, reductions in visits to physicians and better immune system functioning when writing about their own trauma compared to those who undergo control writing [23,24,25]. This data as of yet, however, has not been replicated within the pediatric rheumatology community.

As a pilot study, this study establishes feasibility for the future use of creative writing interventions among pediatric patients with chronic rheumatologic diseases. Recognizing that approximately half of those approached for the study agreed to participate, it is feasible to recruit patients interested in narrative medicine. Over 90% of participants completed pre-study questionnaires and post-participation interviews demonstrating feasibility of the implementation of questionnaires with participation, with 77% completing post-study questionnaires. These completion rates are higher than many other similarly structured interventions, with 71% completion rate for a therapeutic songwriting intervention for those with dementia and their care givers and 73% completion rate for participation in online expressive writing intervention for COVID-19 resilience [26,27].

In the current study, barriers that may have contributed to reduced completion of post-study questionnaires include length of questionnaires and technological difficulties with completion. Future studies can consider shorter questionnaires and/or creating HIPPA-compliant online questionnaire forms for easier accessibility.

Analysis of the questionnaires showed statistical significance between pre- and post-participation PedsQL questionnaires for physical health, without significant differences in the other questionnaires. Given the small sample size, significant differences were not expected. However, the improvement in self-reported physical health suggests an important avenue for future research investigating the potential positive impact of creative writing for patients with chronic rheumatologic diseases. This trend reflects data from randomized controlled trials for patients with fibromyalgia, in which patients had improved self-reported pain, fatigue, and psychological wellbeing following participation in writing exercises [28,29].

As with any study, it is important to address limitations and areas to address in future studies. A potential limitation of this study is that those who participated were self-selected, providing a biased sample. When looking at individuals who chose not to participate in the intervention, it seems narrative medicine interventions may be best offered as an elective rather than mandatory opportunity. An additional limitation is that recruitment of patients relied on recommendations from pediatric rheumatologists of individuals who were interested as well as those who were thought to benefit most from the intervention, which likely led to selection bias. This selection bias is reflected in the patient cohort, noting that all patients were female, and there were few patients with JIA and many with other rare pediatric rheumatology disorders (particularly those with the potential for higher mental health burden). By confining the search for patients attending clinic, there is a potential confounding risk for selection bias as patients with poor visit adherence are less likely to be recruited. However, as this is a pilot study with participants who are willing and available to participate, the risk is accepted as a necessary one. While non-English speaking patients were excluded, it is possible these patients may have significant benefit from narrative medicine interventions and these patients should be considered a target for future narrative medicine interventions. In addition, future studies should consider establishing both a control and experimental group so as to compare outcomes between the two groups. It may be helpful to consider subdivision of future study groups into cohorts with smaller age ranges, which may allow for a better age-appropriate analysis of responses. Another potential limitation is that some participants had exposure to the questionnaires through clinic or prior studies, which may have primed them to answer the questionnaires differently than they would have otherwise. The study did not ask patients if they were being treated by therapists, psychologists or psychiatrists for mental health problems, which may have provided additional insight into the interpretation of the results. The study also did not address whether patients had a controlled or difficult to manage disease, which may be relevant in interpreting data. Finally, this study did not include medication adjustments that may have occurred over the time between pre- and post-questionnaire completion, which may have impacted the results of the study as well.

An important point of reflection for this intervention is the use of an online forum. While an online forum may lack more personal in-person connections, it may allow great accessibility for persons with a chronic illness who are immunosuppressed or disabled. In addition, many patients would not have had alternative modes of connection at the time the study was completed, recognizing that the study occurred early in the pandemic. The pandemic may have impacted questionnaire responses as well, recognizing that pre- and post-surveys may have been completed in different phases of the pandemic. It may be of value to include sessions that are in-person compared to sessions online to understand the potential benefits and limitations of completing this forum online.

In order to maximize benefit and minimize burden for future participants, future studies could consider shorter surveys as well as a greater number of narrative medicine sessions. Additionally, future studies could consider variable lengths of each session to understand what length of time for participation may optimize outcomes while minimizing burden.

In this study, content analysis showed that although participants had different rheumatologic illnesses, patients felt a connection with one another regardless of diagnosis given their shared experiences with the medical world. Chronic illness offered a unified platform with which patients could process and express their stories with one another. Future studies should involve a larger sample size and consider comparing mixed disease processes versus one disease process while monitoring for differences in the writing experience. Additionally, future studies could consider implementing multiple narrative medicine sessions rather than just one to allow for a more robust impact on participants. Participants were appreciative of the opportunity to connect with others, as well as to reflect on their illness experiences.

## 5. Conclusions

In conclusion, creative writing is an intervention that is feasible and acceptable for youth with rheumatologic illnesses. Qualitative analysis of post-participation interviews demonstrated that participants have a strong interest in further narrative medicine involvement, with positive subjective reflections on the experience. Further evaluation is needed to understand if these narrative medicine interventions improve mental and physical symptoms, and children’s attitude towards their illnesses.

## Figures and Tables

**Table 1:** Pre- and post-participation questionnaire medians, including *p*-values. * = Statistically significant.

Questionnaire	Pre-Participation Median [IQR]	Post-Participation Median [IQR]	*p*-Value
PedsQL			
Physical Health	64.95 [56.25–79.96]	78.15 [62.5–93.8]	0.02 *
Psychosocial Health	65 [58.6–80.85]	68.95 [57.1–73.3]	0.3
PSC-17	17 [13–25]	14.5 [12–253]	0.7
PHQ-9	5 [3–13]	6 [4–12]	0.4
CATIS	3.1 [2.7–3.4]	3.24 [2.5–3.8]	0.2

Abbreviations: Peds QL: Pediatric Quality of Life Inventory; PSC-17: Pediatric Symptom Checklist-17; PHQ-9: Patient Health Questionnaire-9; and CATIS: Child Attitude Toward Illness Scale.

## Data Availability

Data is available upon request.
